# DNA Barcoding of Morphologically Characterized Mosquitoes Belonging to the Genus *Mansonia* from the Atlantic Forest and Brazilian Savanna

**DOI:** 10.3390/insects14020109

**Published:** 2023-01-20

**Authors:** Karin Kirchgatter, Lilian de Oliveira Guimarães, Eliana Ferreira Monteiro, Vanessa Christe Helfstein, Juliana Telles-de-Deus, Regiane Maria Tironi de Menezes, Simone Liuchetta Reginato, Carolina Romeiro Fernandes Chagas, Vera Lucia Fonseca de Camargo-Neves

**Affiliations:** 1 Pasteur Institute, São Paulo 01027-000, SP, Brazil; 2 Postgraduate Program in Tropical Medicine, School of Medicine, University of São Paulo, São Paulo 05403-000, SP, Brazil; 3 Nature Research Centre, 08412 Vilnius, Lithuania; 4 Applied Research Department, São Paulo Zoological Foundation, São Paulo 04301-905, SP, Brazil

**Keywords:** *Mansonia*, Brazil, DNA barcode, *COI* sequences, Culicidae

## Abstract

**Simple Summary:**

This study aimed to provide genetic data of *Mansonia*, a mosquito species that is an important vector of viruses and other parasites to both humans and animals. The morphological identification of this species, is quite difficult, even for experienced entomologists, and often requires the assembly of male genitalia, whose structural characters allow for accurate identification of most species, which is not always possible. The DNA sequences obtained in this study can be used for future molecular identifications of this species (DNA barcoding).

**Abstract:**

The identification of mosquito species is necessary for determining the entomological components of disease transmission. However, identification can be difficult in species that are morphologically similar. The cytochrome *c* oxidase subunit I (*COI*) DNA barcode region is considered a valuable and reliable diagnostic tool for mosquito species recognition, including those that belong to species complexes. *Mansonia* mosquitoes are found in forests near swampy areas. They are nocturnal and are highly attracted to light. Hematophagous adult females exhibit aggressive biting behavior and can become infected with and transmit pathogens during their feeding, including some epizootic viruses and avian malaria. In Brazil, twelve *Mansonia* species have been reported. In a recent study from the São Paulo Zoo in Brazil, three morphologically distinct species were collected and identified, namely: *Mansonia* (*Mansonia*) *indubitans*, *Ma.* (*Man.*) *pseudotitillans* and *Ma.* (*Man.*) *titillans.* However, confirmation of these species by molecular identification was unsuccessful due to a lack of *COI* sequences in the GenBank database. Thus, this research aimed to describe the *COI* DNA barcode sequences of some morphologically characterized *Mansonia (Man.)* species from Brazil and to determine their utility in delimiting species collected from the Atlantic Forest and Brazilian Savanna. Accordingly, we provide tools for the genetic identification of species that play a significant role in pathogen transmission in wildlife and potentially humans. We show that the delimitation of *Mansonia* species via five different approaches based on *COI* DNA sequences (BI, NJ, ASAP, bPTP and GMYC) yield basically the same groups identified by traditional taxonomy, and we provide the identification of specimens that were previously identified only up to the subgenus level. We also provide *COI* sequences from two *Mansonia* species that were not previously available in sequence databases, *Ma. wilsoni* and *Ma. pseudotitillans*, and thus contribute to the ongoing global effort to standardize DNA barcoding as a molecular means of species identification.

## 1. Introduction

Mosquito species identification is crucial for determining the entomological components of disease transmission, but it can be difficult in species that are morphologically similar. Moreover, the successful identification of these challenging species groups using morphology is time-consuming, requires rare taxonomic expertise, and is dependent on the integrity of the external characteristics of the specimens (e.g., scales can be lost during collection) [1].

Taxonomic keys are the most common method used for the identification of adult mosquitoes. They involve a stepwise comparison of morphological features, selecting among the ones that fit the described characteristics and eliminating species that do not fit the description, until a conclusion is reached. However, taxonomic keys are complex and have several limitations. For instance, there may be some natural variation between different populations of the same species, or it is simply not possible to distinguish between some species based on external appearance alone [1].

DNA sequences are used as an additional tool for species recognition, including those that belong to species complexes [2]. Herbert and co-workers proposed the use of a 658 base pair (bp) region of the cytochrome *c* oxidase subunit 1 (*COI*) gene as a universal marker to barcode animal life [3]. The *COI* DNA barcode region is considered a valuable and reliable diagnostic tool for studying the genetic structure and diversity of mosquitoes (Diptera: Culicidae) [4,5,6,7].

The Culicidae is divided into two subfamilies, the Anophelinae and the Culicinae. Within the Culicinae, ten tribes are recognized: Aedeomyiini, Aedini, Culicini, Culisetini, Ficalbiini, Hodgesiini, Mansoniini, Orthopodomyiini, Sabethini, and Uranotaeniini [8].

The Mansoniini comprises two genera: *Mansonia* Blanchard, 1901 and *Coquillettidia* Dyar, 1905. These genera usually deposit their eggs directly on the surface of the water or in aquatic vegetation, where the larvae are fixed by their respiratory siphons. Immature forms of these two genera have a spiracular apparatus adapted to perforate the submerged vegetation and obtain oxygen from the tissues of plants [9]. Thus, an abundance of aquatic plants and a reduction in water flow can facilitate the proliferation of these mosquitoes, which are accordingly found in forests near swampy areas. *Mansonia* mosquitoes are nocturnal and are highly attracted to light. Hematophagous adult females exhibit aggressive biting behavior and are often a nuisance to humans [10]. 

The *Mansonia* genus comprises 27 species distributed in two subgenera. The subgenus *Mansonioides* Theobald, 1907 consists of 12 species, ten distributed in Asia and two in Ethiopia [11]. The subgenus *Mansonia* contains 15 neotropical species [10,12,13,14], namely: *Mansonia amazonensis* Theobald, 1901, *Mansonia cerqueirai* Barreto & Coutinho, 1944, *Mansonia chagasi* da Costa Lima, 1935, *Mansonia dyari* Belkin, Heinemann & Page, 1970, *Mansonia flaveola* Coquillett, 1906, *Mansonia fonsecai* Pinto, 1932, *Mansonia humeralis* Dyar and Knab, 1916, *Mansonia iguassuensis* Barbosa, Navarro da Silva & Sallum, 2007, *Mansonia indubitans* Dyar & Shannon, 1925, *Mansonia leberi* Boreham, 1970, *Mansonia pessoai* Barreto & Coutinho, 1944, *Mansonia pseudotitillans* Theobald, 1901, *Mansonia suarezi* Cova Garcia & Sutil Oramas, 1976, *Mansonia titillans* Walker, 1848, and *Mansonia wilsoni* Barreto & Coutinho, 1944. Only *Ma. titillans* and *Ma. indubitans* have a distribution that reaches the southern tip of the Nearctic Region. *Mansonia titillans* is the species with the widest geographical distribution, being found in the United States (Florida, Texas), Mexico, South and Central America, and the Antilles [12].

In Brazil, the *Mansonia* genus is near ubiquitously distributed [12,13], with twelve species currently known in the country: *Ma. amazonensis*, *Ma. cerqueirai*, *Ma. chagasi*, *Ma. flaveola*, *Ma. fonsecai*, *Ma. humeralis*, *Ma. iguassuensis*, *Ma. indubitans*, *Ma. pessoai*, *Ma. pseudotitillans*, *Ma. titillans*, and *Ma. wilsoni* [15].

A recent study conducted at the São Paulo Zoo aimed to identify potential vectors of avian *Plasmodium*. One hundred and eight specimens of *Mansonia* were found and were morphologically identified as three distinct species: *Ma. indubitans*, *Ma. pseudotitillans*, and *Ma. titillans* [16]. To confirm the mosquito species infected with hemosporidian parasites, we conducted molecular identification using DNA barcoding. However, for mosquitoes of the *Mansonia* genus, species identification based on the best close match (BCM) approach was unsuccessful since queries in the GenBank database (hereafter GenBank) returned BCM values far below the threshold (90%) for a successful identification (>99%) [16]. A screening for *COI* sequences of *Mansonia* species in GenBank revealed sequences for *Ma. titillans* (Colombia and Mexico), *Ma. indubitans* (Brazil and Colombia), *Ma. flaveola* (Puerto Rico), *Ma. humeralis* (Argentina), *Ma. dyari* (Mexico and Virgin Islands), and *Ma. amazonensis* (Brazil). Therefore, of the sequences available in GenBank, only two sequences were from Brazil and not even half of the species described in Brazil had sequences deposited in GenBank; no sequences were found for *Ma. cerqueirai*, *Ma. chagasi*, *Ma. fonsecai*, *Ma. iguassuensis*, *Ma. pessoai*, *Ma. pseudotitillans,* and *Ma. wilsoni.*

Although there are few studies exploring the vector potential of *Mansonia* (*Man*.) species, some specimens have been found to be infected with arboviruses and other pathogens. *Mansonia indubitans* is moderately susceptible to infection with four strains of Venezuelan equine encephalitis virus (VEEV) [17,18,19]. *Mansonia titillans* is a species from which both epizootic [20] and enzootic [21] VEEV was isolated and shows an intermediate capacity to become infected with and transmit epizootic viruses [17,22]. Saint Louis encephalitis virus (SLEV) was detected in *Ma. titillans* for the first time in Colombia [23] but was also reported in Argentina [24]. The Bunyamwera serogroup, one of the most important serogroups in the *Orthobunyavirus* genus [25], was also reported in *Ma. titillans* from Argentina [24]. Lastly, the occurrence of avian *Plasmodium* lineages in *Mansonia* mosquitoes from Brazil has been reported in two species (*Ma. titillans* and *Ma. pseudotitillans*) [26]. More recently and as mentioned before, the avian pathogens *Plasmodium nucleophilum* and *Haemoproteus* (*Parahaemoproteus*) sp. were observed in two specimens in Brazil, *Mansonia indubitans* and *Mansonia* (*Man*.) sp. [16].

Studies on insect genetics are important for improving vector control measures and aim to prevent or reduce epidemic impacts. Thus, this research aimed to describe the *COI* DNA barcode sequences of some morphologically characterized *Mansonia* (*Man*.) species from Brazil and to determine their utility in delimiting species collected in the Atlantic Forest and Brazilian Savanna. Accordingly, we provide tools for the genetic identification of species that play a significant role in pathogen transmission in wildlife and potentially humans.

## 2. Materials and Methods

### 2.1. Mosquito Sampling and Handling

**Figure 1:**
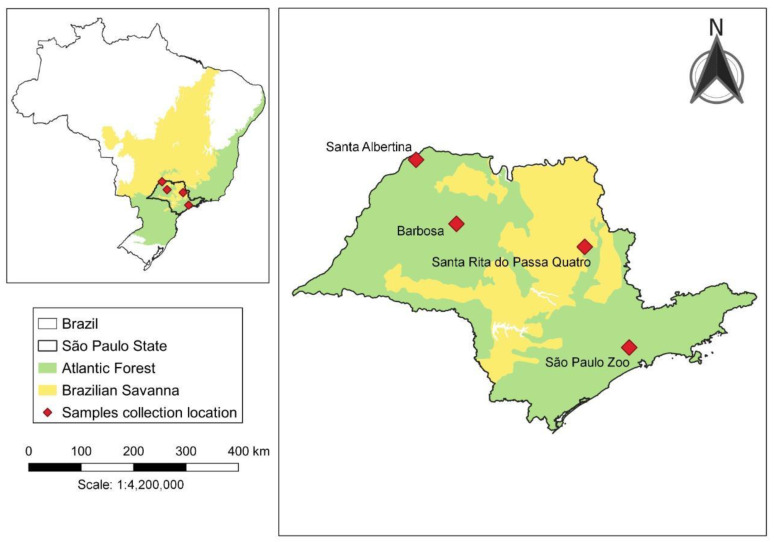
Location of municipalities in the State of São Paulo, Brazil, where *Mansonia* specimens were collected.

Female mosquitoes were collected at four study sites in the State of São Paulo, Brazil. Study sites and years are shown in Figure 1 and Table 1. Santa Albertina (20°01′55″ S, 50°43′40″ W), Barbosa (21°16′00″ S, 49°56′57″ W), and Santa Rita do Passa Quatro (21°42′36″ S, 47°28′40″ W) are municipalities located in the northwest, north-northwest, and northeast regions of the State of São Paulo, respectively. The São Paulo Zoo (23°39′03″ S, 46°37′14″ W) is in the city of São Paulo, the capital of the State with the same name, and situated in its southeast region (Figure 1).

Mosquitoes were collected using CDC (Center for Disease Control) light traps [27] baited with CO_2_ (dry ice) for 12 h. The traps were set at dusk and removed a few hours after sunset or with Nasci aspirator for 2 h, from 9:00 to 11:00 a.m. [28]. Part of the mosquitoes were killed with chloroform vapor and part were killed in liquid nitrogen and transported to the laboratory for taxonomic identification using morphological taxonomic keys [10,29,30]. Mosquitoes were individually stored at −20 °C in 1.5 mL plastic tubes sealed with parafilm before molecular processing.

The photomicrographs were performed under a Leica M205C stereomicroscope, with images being captured with an attached Leica DFC320 digital camera and processed with the FusionOptics technology that provides a 3D image in Leica Application Suite 3.7 (Leica Microsystems, Wetzlar, Germany). The maxillary palpus and proboscis lengths were measured using this system.

### 2.2. Genomic DNA Extraction and PCR Amplification of Mitochondrial Gene Fragments

Collected mosquitoes were transferred to a Master Mix lysis buffer [200 µL Nuclei Lysis Solution, 50 µL EDTA (Ethylene Diamine Tetraacetic Acid) 0.5 M (pH 8.0), 20 µL proteinase K (20 mg/mL), and 5 µL RNase A Solution] and thereafter triturated using FastPrep-96 (MP Biomedicals, Solon, OH, USA) in combination with two 1.4 mm ceramic beads (MagNA Lyser Green Beads-Roche Molecular Systems) coated with 6.35 mm zirconium oxide (MP Biomedicals). The trituration process was conducted for 3 min at 1800 rpm. Samples were then centrifuged at room temperature for 5 min, at 14,000 rpm. DNA was extracted using the Wizard SV 96 Genomic DNA Purification System (Promega) according to manufacturer instructions. Finally, extracted DNA was eluted in 100 µL of nuclease-free water and stored at −20 °C until analysis.

A fragment of 710 base pairs (bp) of the barcode region of the mitochondrial *COI* gene was amplified by PCR using the primers LCO1490/HCO2198 [31], following the protocol proposed by Ruiz et al. [32].

### 2.3. Sequencing, Alignment, and Sequence Analysis

PCR products were directly sequenced in both directions by means of a BigDye Terminator v3.0 Cycle Sequencing Kit in an ABI Genetic Analyzer (Applied Biosystems^®^, Foster City, CA, USA); corresponding flanking primers were used. Sequences were aligned with reference sequences (Table 2) using Clustal W [33], inspected, and edited within MEGA version X [34]. Obtained sequences were deposited within the GenBank database (OQ120978-OQ121013).

For the phylogenetic analysis, an alignment matrix was prepared. The matrix consisted of 36 *COI* sequences from the collected specimens morphologically identified as: *Ma. humeralis*, *Ma*. *pseudotitillans*, *Ma. titillans*, *Ma. wilsoni*, *Ma. indubitans*, and *Mansonia* (*Man*.) sp. (Table 1). Additionally, the matrix included another 41 *COI* sequences of *Mansonia* (*Man*.) species from other Neotropical areas that were retrieved from GenBank (Table 2), and only sequences with >609 bp were used.

**Table 1:** Collected female *Mansonia (Man.)* spp., according to collection year, period, method, strata, and sites.

Sample ID	Collection Year	Period	Method	Strata	Collection Site	Species (According to Taxonomic Keys)
A290E	2019	D	Nasci	G	Santa Albertina	*Mansonia humeralis*
A290K	2019	D	Nasci	G	Santa Albertina	*Mansonia humeralis*
A290L	2019	D	Nasci	G	Santa Albertina	*Mansonia humeralis*
A290M	2019	D	Nasci	G	Santa Albertina	*Mansonia humeralis*
A290W	2019	D	Nasci	G	Santa Albertina	*Mansonia titillans*
A5725	2020	N	CDC	G	Santa Rita do Passa Quatro	*Mansonia* (*Man.*) sp.
A613	2019	D	Nasci	G	Barbosa	*Mansonia humeralis*
A613B	2019	D	Nasci	G	Barbosa	*Mansonia humeralis*
B173	2020	N	CDC	C	São Paulo Zoo	*Mansonia wilsoni*
B189	2020	N	CDC	C	São Paulo Zoo	*Mansonia* (*Man.*) sp.
B240	2020	N	CDC	C	São Paulo Zoo	*Mansonia* (*Man.*) sp.
B245	2020	N	CDC	C	São Paulo Zoo	*Mansonia* (*Man.*) sp.
B378	2020	N	CDC	C	São Paulo Zoo	*Mansonia wilsoni* aff
B556	2020	N	CDC	C	São Paulo Zoo	*Mansonia wilsoni* aff
B615	2020	N	CDC	G	São Paulo Zoo	*Mansonia* (*Man.*) sp.
B83	2020	N	CDC	C	São Paulo Zoo	*Mansonia* (*Man.*) sp.
B926	2020	N	CDC	C	São Paulo Zoo	*Mansonia wilsoni* aff
Zoo044	2015	N	CDC	G	São Paulo Zoo	*Mansonia* (*Man.*) sp.
Zoo252	2015	N	CDC	G	São Paulo Zoo	*Mansonia* (*Man.*) sp.
Zoo555	2015	N	CDC	G	São Paulo Zoo	*Mansonia* (*Man.*) sp.
Zoo634	2015	N	CDC	G	São Paulo Zoo	*Mansonia* (*Man.*) sp.
Zoo683	2015	N	CDC	G	São Paulo Zoo	*Mansonia* (*Man.*) sp.
Zoo684	2015	N	CDC	G	São Paulo Zoo	*Mansonia* (*Man.*) sp.
Zoo685	2015	N	CDC	G	São Paulo Zoo	*Mansonia* (*Man.*) sp.
Zoo686	2015	N	CDC	G	São Paulo Zoo	*Mansonia* (*Man.*) sp.
Zoo798	2015	N	CDC	G	São Paulo Zoo	*Mansonia* (*Man.*) sp.
Zoo799	2015	N	CDC	G	São Paulo Zoo	*Mansonia indubitans*
Zoo800	2015	N	CDC	G	São Paulo Zoo	*Mansonia* (*Man.*) sp.
ZooB050	2015	N	CDC	G	São Paulo Zoo	*Mansonia pseudotitillans*
ZooB252	2015	N	CDC	G	São Paulo Zoo	*Mansonia* (*Man.*) sp.
ZooB253	2015	N	CDC	G	São Paulo Zoo	*Mansonia pseudotitillans*
ZooB365	2015	N	CDC	G	São Paulo Zoo	*Mansonia pseudotitillans*
ZooB383	2015	N	CDC	G	São Paulo Zoo	*Mansonia pseudotitillans*
ZooB591	2015	N	CDC	G	São Paulo Zoo	*Mansonia* (*Man.*) sp.
ZooB592	2015	N	CDC	G	São Paulo Zoo	*Mansonia pseudotitillans*
ZooB797	2015	N	CDC	G	São Paulo Zoo	*Mansonia* (*Man.*) sp.

Note: Period: D = day and N = night; Strata: C = canopy and G = ground. (*Man*.) = abbreviation of the subgenus *Mansonia* according to Reinert [35].

**Table 2:** GenBank accession numbers of the reference *Mansonia (Man.)* sequences used in the study.

Species	Country Source	#GenBank
*Mansonia indubitans*	Brazil (Caatinga)	MH118158
*Mansonia indubitans*	Colombia	MN997669-MN997672
*Mansonia flaveola*	Puerto Rico	JX260065
*Mansonia humeralis*	Argentina	MW363430-MW363432
*Mansonia titillans*	Colombia	KT766533
*Mansonia titillans*	Colombia	KY859898-KY859902 MN997665-MN997667
*Mansonia titillans*	Mexico	MN968219, MN968225, MN968231, MN968233, MN968240, MN968241, MN968244, MN968259, MN968266, MN968270, MT999303
*Mansonia dyari*	Mexico	MN968222, MN968243, MN968246, MN968251, MN968254, MN968264, MN968268, MN968272, MN968273, MN968274
*Mansonia dyari*	Virgin Islands	MN129182
*Mansonia amazonensis*	Brazil	MK575483

Phylogenetic tree reconstruction was performed using the Bayesian approach implemented in MrBayes v3.2.2 [36]. This phylogenetic tree was built with the aim of obtaining support values for the taxa where genetic clusters may represent new or cryptic species; it was not our objective to infer phylogenetic relationships between the species analyzed. This Bayesian inference (BI) was conducted with two Markov Chain Monte Carlo (MCMC) chains run simultaneously for 3 million generations, sampling 1 in every 300 trees. After a burn-in of 25%, the remaining 15,002 trees were used to generate a 50% majority-rule consensus tree. The standard deviation of the split frequencies between runs (<0.01) and the effective sample size was monitored to ensure stability, convergence, and correct mixing of the chains. The result of the analysis was visualized using FigTree version 1.4.4 [37]. The topology was rooted using a sequence obtained for *Aedes aegypti* (KX420454). Another tree reconstruction was performed by the Neighbor-Joining (NJ) method using MEGA version X [34] and Kimura-2 Parameter distances. Branch supports of NJ trees were assessed by bootstrapping with 1000 replicates. MEGA version X [34] was also used to compute intraspecific (mean distance within group) and interspecific (mean distance between groups) sequence divergence using the Kimura-2 parameter distance model [38].

### 2.4. Species Delimitation

The use of DNA barcodes for delimiting species into molecular operational taxonomic units (MOTUs) includes a series of strategies that use a combination of laboratory and bioinformatics methods [39]. Beside BI and NJ, three more approaches were used to establish the species delimitation. First, the Assemble Species by Automatic Partitioning (ASAP) was run on a web server (https://bioinfo.mnhn.fr/abi/public/asap/) (accessed on 3 October 2022) using Kimura (K80) ts/tv 2.0; the lower score (1.5) was considered the better partition [40]. Second, the Poisson Tree Processes method (bPTP) [41] based on the unrooted binary maximum likelihood (ML) tree (GTR+G) was used and obtained with MEGA version X [34] and implemented on a web server (https://species.h-its.org/ (accessed on 3 October 2022)) applying default settings. The bPTP adds Bayesian support (BS) values to delimited species on the input tree; the higher the BS, the more likely it is that the taxa forming the node belong to the same species. Third, the Generalized Mixed Yule Coalescent model (GMYC) [42] based on an ultrametric tree resulting from a single locus was used and was run on a web server (http://species.h-its.org/gmyc/ (accessed on 3 October 2022)) using default parameters.

## 3. Results

### 3.1. Morphological Assessment

We identified a total of 17 specimens to the species level: six *Ma. humeralis*, five *Ma*. *pseudotitillans*, one *Ma. titillans*, four *Ma. wilsoni*, and one *Ma. indubitans.* Nineteen specimens were identified only up to the subgenus level (Table 1). Below, we list and provide photographs of some characteristics used in the identification of *Mansonia* species analyzed in this study that we consider decisive, subjective, or difficult to visualize.

#### 3.1.1. Maxillary Palpus

**Figure 2:**
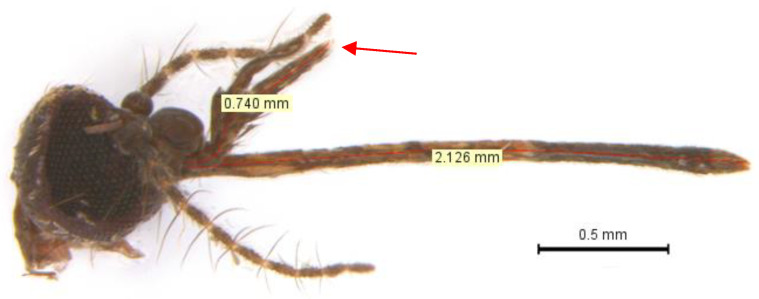
Head of *Mansonia pseudotitillans* (ID ZooB592) showing (arrow) maxillary palpus (0.740 mm) about 0.29 of the proboscis total length (2.126 mm).

**Figure 3:**
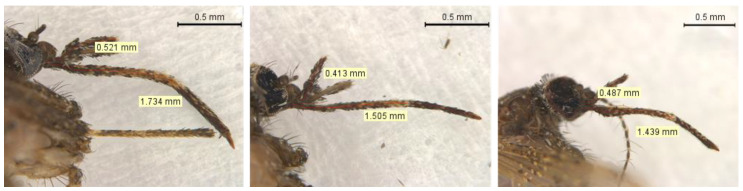
Head of three specimens of *Mansonia pseudotitillans* (IDs A7206A, A7206B e A7208) showing maxillary palpus with 0.33, 0.36 and 0.29 of the total proboscis length.

*Mansonia indubitans* and *Ma. pseudotitillans* are difficult to differentiate due to the similarity of their maxillary palpus. A maxillary palpus with less than one fourth proboscis length (0.25) are characteristics of *Ma. indubitans*, while a maxillary palpus with more than 0.3 of the proboscis length are attributed to *Ma. pseudotitillans* [10,43]. Using morphometry, we were able to confirm this feature in the identification of different *Ma. pseudotitillans* individuals, although in some cases the measure was slightly below 0.3 (Figure 2 and Figure 3).

#### 3.1.2. Spines on the Abdominal Tergite VII and Suprawing Scale

**Figure 4:**
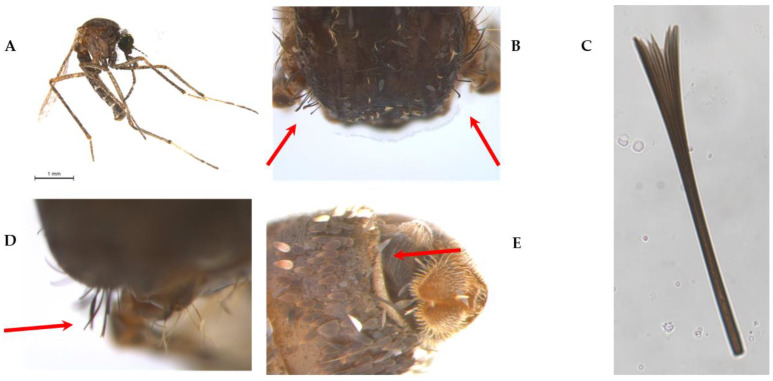
(**A**) *Mansonia titillans* (ID A290W). (**B**–**D**) Magnifications showing suprawing scale (arrows). (**E**) Abdominal tergite VII with spines.

Two further morphological characters that were examined to identify the female *Mansonia* specimens are the suprawing scale and the potential presence of spines on the abdominal tergite VII (Figure 4).

The taxonomic key of Forattini 2002 [10] uses the following as a differentiating criterion of *Mansonia titillans* (Figure 4A): the presence of suprawing scales with a simple apex rather than suprawing scales with a forked apex, for *Ma. pseudotitillans*, *Ma. indubitans*, or *Ma. dyari.* However, we consider this characteristic to be difficult to identify (Figure 4B–D), even using magnification of a suprawing scale (Figure 4C).

According to the taxonomic keys of Forattini 2002 [10] and Assumpção 2009 [30], *Ma. indubitans* do not present spines on the abdominal tergite VII. However, the authors disagree about the presence of the same character in *Ma. pseudotitillans*: the key by Forattini 2002 [10] states that this species does not have spines in the abdominal segment VII and both the key and the description by Assumpção 2009 [30] mention spines in that segment, as well as Forattini 2002 [10] for *Ma. titillans* (Figure 4E).

### 3.2. Sequence Analysis and Species Delimitation

**Figure 5:**
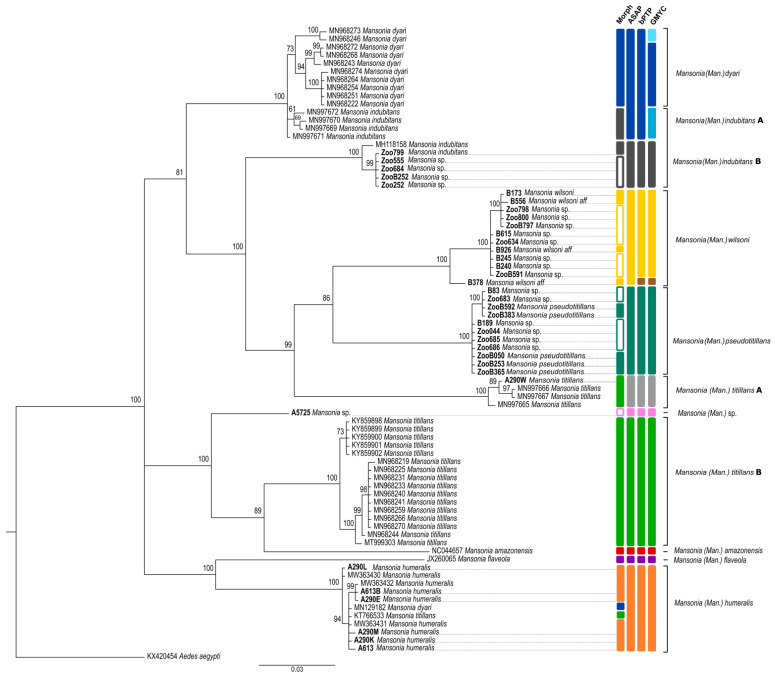
Bayesian inference tree based on the cytochrome *c* oxidase I (*COI*) barcoding region of *Mansonia* (*Man*.) species. This analysis involved 77 nucleotide sequences. *Aedes aegypti* (KX420454) was used as an outgroup. There was a total of 641 positions in the final dataset. Average standard deviation of the split frequencies was 0.004342. Posterior probability values are shown for each clade. Specimens colored blank in the morphology column were not morphologically identified to species level due to a lack of important characters. Sequences obtained in this study are given in bold.

The 36 *COI* sequences obtained for the present study (with sequences ranging from 559 to 658 bp) represented five different *Mansonia* species. Of these, two sequences were, for the first time, linked to morphologically identified specimens: *Ma. wilsoni* and *Ma. pseudotitillans*. The results of the analyses using these DNA sequences are shown in Figure 5. Bayesian analysis of the obtained *COI* sequences resulted in clade topologies that corroborate with the morphologically identified species (except for MN129182 and KT766533). However, for some species, the tree did not group all sequences into the same clade. For example, *Ma. indubitans* appears polyphyletic and is separated into two different clades (Figure 5). Further, the *Ma. wilsoni* and *Ma. pseudotitillans* clades were split into subclades, indicating a cryptic diversity in this species collected in the São Paulo State.

The BI tree shows that sequences collected from Brazil in this study basically clustered in two strongly supported clades (Figure 5). The first one (*Ma. humeralis* clade) was formed by six sequences from Brazil and five different GenBank sequences (mainly *Ma. humeralis* sequences). The second clade of sequences from Brazil was split into four subclades encompassing: (i) sequences from specimens identified as *Ma. titillans* (clade *Ma. titillans* A); (ii) one monophyletic clade with all our *Ma. pseudotitillans* sequences, but without any GenBank reference sequence; (iii) one monophyletic clade with all *Ma. wilsoni* sequences, but again without any GenBank reference; and (iv) one sequence of the polyphyletic *Ma. indubitans* from GenBank that grouped with five *Ma. indubitans* sequences obtained in the Sao Paulo State (clade *Ma. indubitans* B) (Figure 5).

The NJ tree further supported the species identities from Brazil. Each species was represented by well supported clades (>98% bootstrap support), confirming the morphological identification (Figure S1 in Supplementary Material).

As shown in Table 3, the mean intraspecific K2P distances for all the species were less than 2%. The maximum distance was seen among the sequences of *Ma. dyari* which was 1%, while *Ma. wilsoni* and *Ma. pseudotitillans* reported the lowest mean intraspecific distance of 0.2%. The interspecific distances ranged from 9.6% between *Ma. amazonensis* and *Ma. titillans* B to 18.3% between *Ma. titillans* A and *Ma. flaveola.* Interspecific distances obtained between *Ma. titillans* A and B and *Ma. indubitans* A and B were 13.4% and 10.9%, respectively. *Mansonia* sp. A5725 showed 7.3% interspecific distance with *Ma. titillans* B, while *Mansonia wilsoni* aff B378 presented 2.1% interspecific distance with the *Ma. wilsoni* group. *Aedes aegypti* showed the highest values, ranging from 14.7 to 18.9% (Table 3).

Each of the species delimitation methods (ASAP, bPTP and GMYC) obtained a result that corroborates with the Bayesian and Neighbor-joining analyses, with some slight variations (Figure 5 and Figures S1–S4 in Supplementary Material). A correspondence in ASAP (Figure S2) and bPTP (Figure S3) algorithms was observed for all the species, which were split into ten and eleven MOTUs, respectively, with the only difference being that taxon B378 was considered as a different species by bPTP but grouped with *Ma. wilsoni* by ASAP (score 1.5) (Figure 5). On the other hand, GMYC analysis resulted in 13 clusters (Figure S4), where specimen B378 was also considered as a different species. In contrast, however, some barcodes of *Ma. indubitans* (clade *Ma. indubitans* A) and *Ma. dyari* were merged into a single MOTU (Figure 5) in ASAP and bPTP algorithms, while they were split into three MOTUs using GMYC (Figure S4).

Sequences of *Ma. titillans* formed two MOTUs using all algorithms. However, *Ma. wilsoni, Ma. pseudotitillans*, and *Ma. humeralis* were strongly supported as a monophyletic species by Bayesian posterior probability (100 BPP) (Figure 5), with a clade formed by specimens from Argentina and Brazil in the case of *Ma. humeralis* (Figure 5). Likewise, the ASAP, bPTP, and GMYC approaches clustered *Ma. wilsoni* and *Ma. pseudotitillans* into a single molecular species (Figure 5 and Figures S2–S4).

## 4. Discussion

Morphological identification of *Mansonia* species is challenging and individuals from this genus are notoriously difficult to distinguish unless the male genitalia—which are needed to differentiate some morphological characters—are examined under a microscope. This study sought to determine the utility of DNA barcoding in delimiting species from the genus *Mansonia* collected in the Atlantic Forest and Brazilian Savanna. The total number of MOTUs within the same taxon varied very little and was independent of the model used to partition the *COI* data, since all models recovered all eight species identified by traditional morphology. Each of the molecular delimitation methods used (ASAP, bPTP, GMYC, NJ and BI) showed highly supported clusters in the identified operational taxonomic units with few differences in their topologies. *Mansonia indubitans* and *Ma. titillans* were recovered as polyphyletic, while *Ma. humeralis*, *Ma. wilsoni,* and *Ma. pseudotitillans* were supported as a monophyletic species according to all methodologies.

A value of 3% in the interspecific distance between *COI* sequences is considered as the threshold in differentiating species [2,3] and this has been applied in many mosquito studies [1,4,5]. Here, the intraspecific distances of all the species identified was less than 3% (ranging from 0.2 to 1%), while the interspecific distances were above 3% (ranging from 9.6 to 18.3%). The interspecific distances confirmed two groups of *Ma. titillans* and *Ma. indubitans,* with values of 13.4% and 10.9%, respectively. They also pointed to a different species (among those analyzed here) in *Mansonia* sp. A5725 (closest interspecific distance was 7.3% with *Ma. titillans* B), a result shared by ASAP, bPTP, and GMYC. Moreover, the interspecific distance of 2.1% supported *Mansonia wilsoni* aff B378 within the *Ma. wilsoni* group, a result corroborated by morphology and ASAP.

Of the 58 sequences with morphological identification to species, only six were not correctly positioned by ASAP, bPTP, and GMYC. These were: two potentially morphologically misidentified GenBank sequences (MN129182 and KT766533) that grouped with *Ma. humeralis* sequences and four *Ma. indubitans* sequences from Colombia that clustered with the sequences of *Ma. dyari* from Mexico. It is worth mentioning that the sequences MN129182 and KT766533 were also identified as *Ma. humeralis* using the BOLD platform (Barcode of Life Data system) using the option “Species Level Barcode Records” (http://www.boldsystems.org/index.php/IDS_OpenIdEngine) (accessed on 3 October 2022).

In fact, in the original description, *Ma. dyari* was considered a taxon morphologically close to *Ma. indubitans* due to the absence of spines on the posterior margin of tergite VII and by the presence of evenly spaced spines on tergite VIII [44]. However, the authors insisted that the “*indubitans*” of the Caribbean area are different from those in South America and named them as *Ma. dyari* [44]. Based on morphologic and phylogenetic data, *Ma. dyari* and *Ma. indubitans* were also grouped into a clade (with 76% bootstrap) [14]. More recently, this cluster was also found by DNA barcoding analysis, pointing to the occurrence of cryptic speciation within *Ma. dyari* [45]. Nevertheless, in our analysis, the sequences of *Ma. indubitans* collected from Brazil clustered in a clade distinct from the one mentioned above (*Ma*. *dyari/indubitans* A). The clade named *Ma. indubitans* B comprised one *Ma. indubitans* sequence from São Paulo Zoo and four *Mansonia* sp. sequences that were clustered with a sequence from the *Ma. indubitans* collected in the Brazilian Caatinga (MH118158). Thus, we believe these unidentified specimens from São Paulo Zoo were *Ma. indubitans,* although some characteristics necessary to confirm morphological identification were missing. It is important to note that our GMYC analysis positioned these two clades (*Ma. indubitans* A and B) as a monophyletic group, including the *Ma. dyari* sequences.

Adult female specimens of *Ma. indubitans* and *Ma. pseudotitillans* are also difficult to differentiate because one important character that distinguishes them, according to the key of Forattini 2002 [10], may vary between individuals of the same species: the proportion of maxillary palpus in relation to the proboscis. However, the divergence between the keys of Forattini 2002 [10] and Assumpção 2009 [30] regarding the presence of spines on the abdominal tergite VII may result in misidentification. Here, using morphometry and high-magnification photomicrographs, we were able to identify *Ma. pseudotitillans* according to Forattini 2002 [10], using the proportion of maxillary palpus in relation to the proboscis and the absence of spines on the abdominal tergite VII. Additionally, we have generated the first *COI* sequences of this species.

In contrast, another important distinctive characteristic is the presence of suprawing scales with a simple apex in *Ma. titillans* rather than suprawing scales with a forked apex in *Ma. pseudotitillans*, *Ma. indubitans,* and *Ma. Dyari* [10]. In this case, even using high magnification photomicrographs, we were unable to confirm this characteristic. However, using the *COI* sequences, these species were separated into strongly supported clades and additionally grouped with individuals of the same species previously identified in other studies.

*Mansonia titillans* specimens were recovered as polyphyletic groups according to all methodologies used, where two clades were obtained, named here as *Ma. titillans* A and *Ma. titillans* B. Comparable results have been found in other studies [45]. However, the position of these two clades was different among the methods, positioning as sister clades in GMYC and bPTP approaches. In this study, taxon A5725 was identified as a different species of *Ma. titillans* according to all the approaches but, using the BOLD platform and the option “Species Level Barcode Records”, the *COI* sequence was matched to *Ma. titillans* and was considered as a reliable identification.

Finally, it is important to mention that there are no *Ma. iguassuensis* or *Ma. fonsecai* sequences in the available databases and, despite this species having already been reported in the State of São Paulo [13,46], we did not morphologically identify specimens of this species from our sampling sites. Thus, it is possible that one of the two clades of *Ma. titillans* and one of the two clades of *Ma. indubitans* are, in fact, *Ma. iguassuensis* and *Ma. fonsecai*, respectively. *Mansonia iguassuensis* has often been misidentified as *Ma. titillans* and, due to the morphological similarities between the adults [13,46], *Ma. fonsecai* has been considered a junior synonym of *Ma. indubitans*. New integrative studies combining morphological and molecular analyses are needed to identify the *COI* sequences of these species.

To know the culicid fauna of a given locality, it is also important to identify male specimens through the structure of their genitalia, as well as immature forms [47]. However, it is not always possible to collect male specimens or immature forms. For this reason, in our work, we use *COI* sequences and thus show that these species can be separated into strongly supported clades. Our Bayesian analysis yielded trees with well-supported internal branches (≥90), resolving six out of the eight taxa as monophyletic groups. We provided sequences of the *COI* gene from two *Mansonia* species that were not previously available in sequence databases, *Ma. wilsoni* and *Ma. pseudotitillans*, thereby contributing to the ongoing global effort to standardize DNA barcoding as a molecular means tool for species identification by the Consortium for the Barcode of Life (CBOL). The use of integrated systematics, combining DNA barcodes and phylogenetic inferences, allows us to refine taxonomic identification and better understand the genetics and distribution of mosquito species.

## 5. Conclusions

Our findings show that *Mansonia* species delimitation via the BI, NJ, ASAP, bPTP, and GMYC approaches yields basically the same groups as those identified by traditional taxonomy, and additionally provides the species identification of specimens classified only up to the subgenus level. Morphological taxonomy combined with molecular taxonomy, as performed for many specimens in this study, makes species identification more consistent. However, morphological identification requires specialist taxonomic knowledge, is time consuming, needs well-preserved specimens, and depends on the subjective interpretation of measures in terms of relative sizes. Here, we show that the use of *COI* sequences is a useful tool to identify morphologically similar species of the subgenus *Mansonia* in the State of São Paulo, overcoming the difficulties encountered when using traditional taxonomy alone.

## Figures and Tables

**Table 3:** Mean interspecific (below the diagonal) and intraspecific (along the diagonal) distances for *COI* sequences. Distances were calculated using the Kimura 2-parameter distance algorithm.

	Species	1	2	3	4	5	6	7	8	9	10	11	12	13
1	*Ma. wilsoni*	0.002												
2	*Ma. humeralis*	0.161	0.003											
3	*Ma. titillans* A	0.148	0.158	0.005										
4	*Mansonia* sp. A5725	0.148	0.126	0.142										
5	*Ma. pseudotitillans*	0.112	0.149	0.132	0.153	0.002								
6	*Ma. wilsoni* aff B378	0.021	0.149	0.135	0.138	0.103								
7	*Ma. flaveola*	0.160	0.136	0.183	0.149	0.151	0.155							
8	*Aedes aegypti*	0.152	0.165	0.189	0.147	0.172	0.150	0.174						
9	*Ma. titillans* B	0.143	0.140	0.134	0.073	0.142	0.134	0.151	0.164	0.005				
10	*Ma. indubitans* B	0.124	0.134	0.133	0.120	0.112	0.113	0.155	0.155	0.126	0.003			
11	*Ma. dyari*	0.124	0.139	0.123	0.108	0.118	0.111	0.144	0.137	0.107	0.110	0.010		
12	*Ma. indubitans* A	0.124	0.133	0.120	0.105	0.119	0.111	0.148	0.139	0.108	0.109	0.013	0.004	
13	*Ma. amazonensis*	0.140	0.154	0.148	0.104	0.139	0.133	0.162	0.162	0.096	0.128	0.127	0.122	

## Data Availability

The data presented in this study are available in Supplementary Materials and the GenBank database (https://www.ncbi.nlm.nih.gov/genbank/ (accessed on 3 October 2022)) (GenBank #OQ120978-OQ121013).

## Supplementary Materials

The following supporting information can be downloaded at: https://www.mdpi.com/article/10.3390/insects14020109/s1, Figure S1: Neighbor-Joining tree based on the *COI* barcoding region of *Mansonia* species; Figure S2: *COI* gene species delimitation of *Mansonia* species by ASAP; Figure S3: *COI* gene species delimitation of *Mansonia* species by bPTP; Figure S4: *COI* gene species delimitation of *Mansonia* species by GMYC.
