# Supplementary material for: DNA Barcoding of Morphologically Characterized Mosquitoes Belonging to the Genus Mansonia from the Atlantic Forest and Brazilian Savanna
Source: Insects. 2023 Jan 20;14(2):109. doi: 10.3390/insects14020109 (PMC9964216; doi:10.3390/insects14020109)

#Result of GMYC species delimitation

method: single
likelihood of null model: 724.6707
maximum likelihood of GMYC model: 877.8727
likelihood ratio: 306.4039
result of LR test: 0***

number of ML clusters: 13
confidence interval: 13-13

number of ML entities: 33
confidence interval: 33-33

threshold time: -1e-06

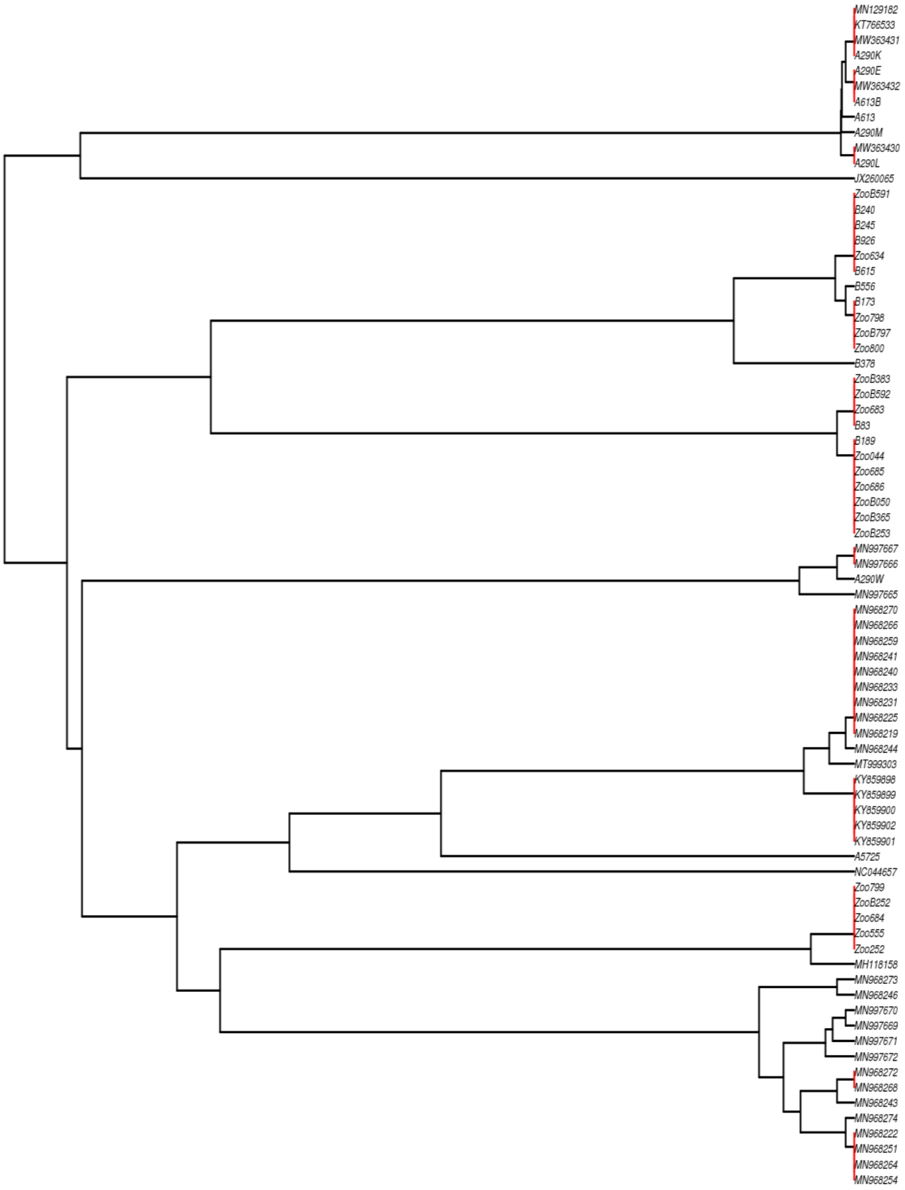

Supplement: Supplementary file 1 [file insects-14-00109-s001.zip › S4_GMYC_Mansonia.pdf]
