# Supplementary material for: DNA Barcoding of Morphologically Characterized Mosquitoes Belonging to the Genus Mansonia from the Atlantic Forest and Brazilian Savanna
Source: Insects. 2023 Jan 20;14(2):109. doi: 10.3390/insects14020109 (PMC9964216; doi:10.3390/insects14020109)
Supplement: Supplementary file 1 [file insects-14-00109-s001.zip › S1_NJ.pdf]

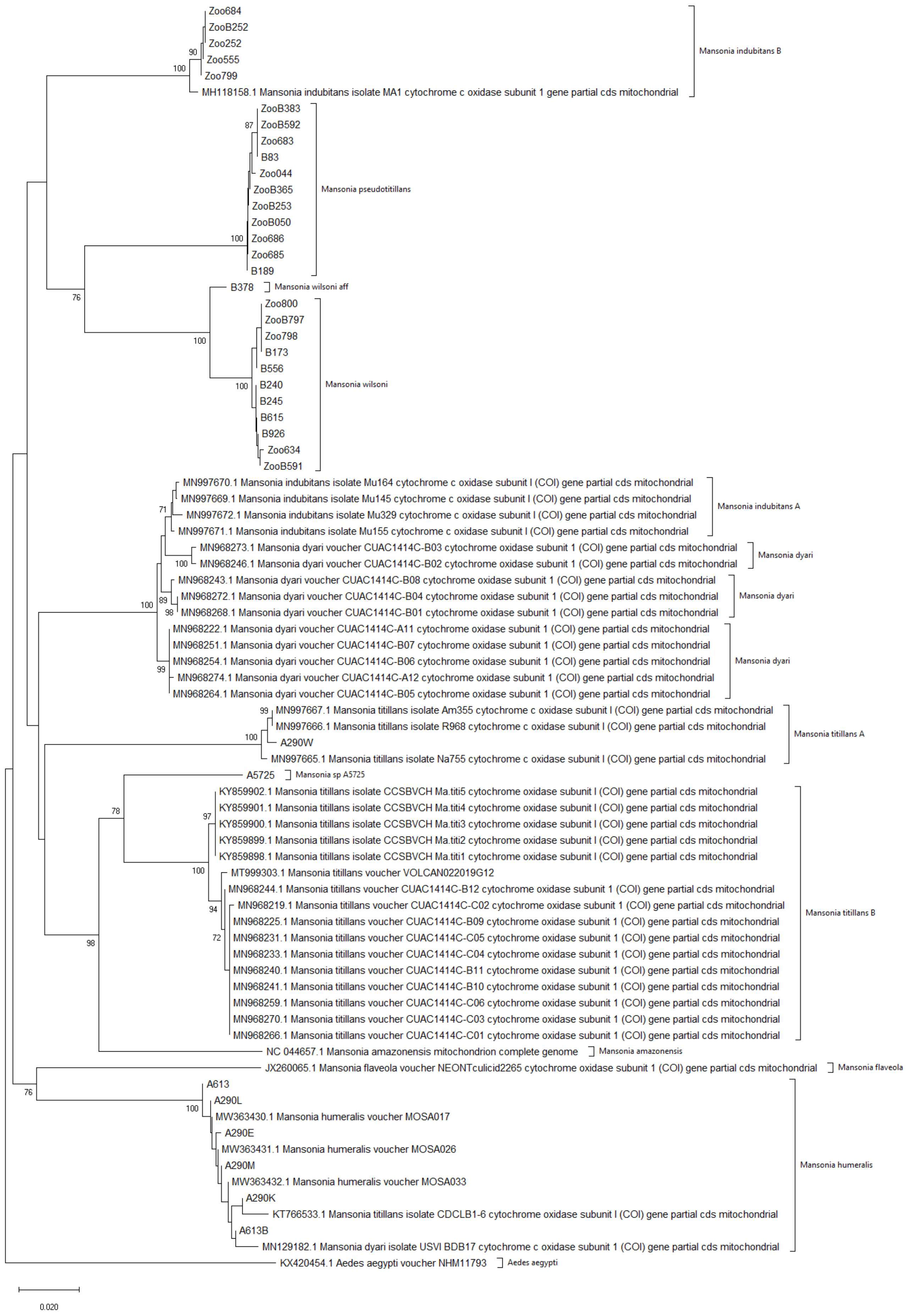

Figure S1. Neighbor-Joining tree based on the *COI* barcoding region of *Mansonia* species. This analysis involved 78 nucleotide sequences with a total of 658 positions in the final dataset. The percentage of replicate trees in which the associated taxa clustered together in the bootstrap test (1000 replicates) are shown next to the branches. Only nodal support > 70% is shown.
