# Supplementary figures and images for: DNA Barcoding of Morphologically Characterized Mosquitoes Belonging to the Genus Mansonia from the Atlantic Forest and Brazilian Savanna
Source: Insects. 2023 Jan 20;14(2):109. doi: 10.3390/insects14020109 (PMC9964216; doi:10.3390/insects14020109)

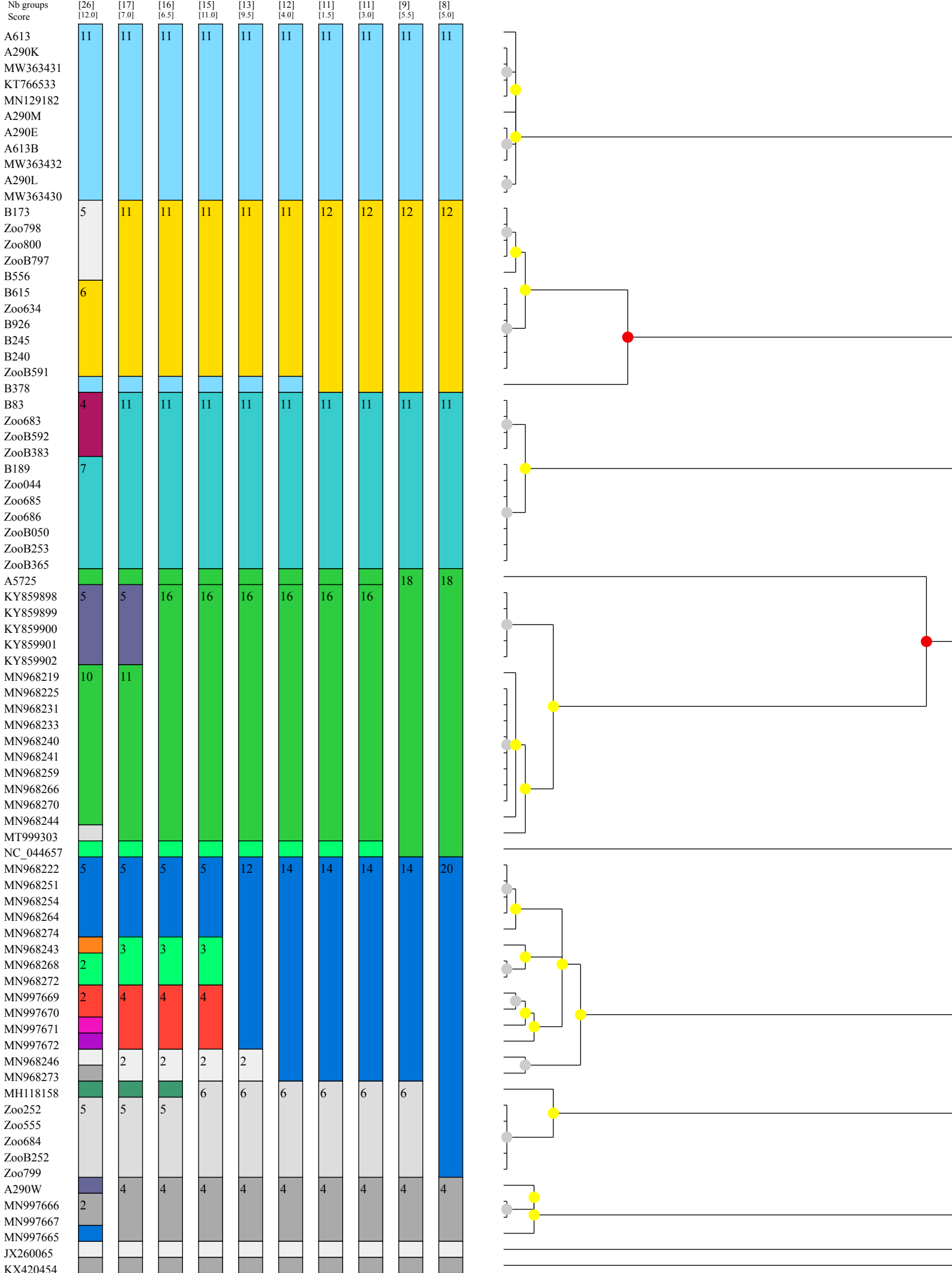

Supplement: Supplementary file 1 [file insects-14-00109-s001.zip › S2_ASAP_Mansonia.pdf]

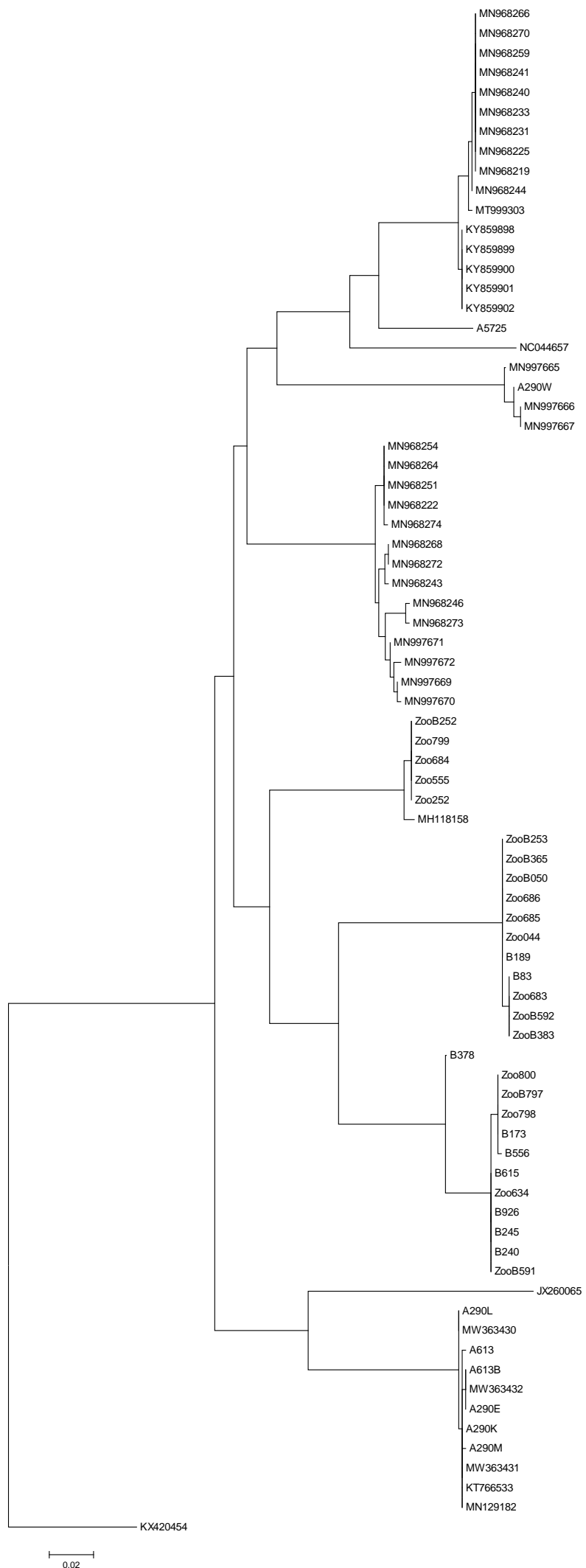

Supplement: Supplementary file 1 [file insects-14-00109-s001.zip › S3_bPTP_Mansonia.pdf]
